# Engaging young people on antimicrobial resistance (AMR): rethinking AMR education for sustainable public health

**DOI:** 10.1093/jacamr/dlag055

**Published:** 2026-04-22

**Authors:** A McKirdy, N A Q Tham, J E le Jeune d'Allegeershecque, D Hale, A Dallman-Porter, K Grailey

**Affiliations:** Fleming Initiative, Institute of Global Health Innovation, Imperial College London, London, UK; Fleming Initiative, Institute of Global Health Innovation, Imperial College London, London, UK; Fleming Initiative, Institute of Global Health Innovation, Imperial College London, London, UK; Fleming Initiative, Institute of Global Health Innovation, Imperial College London, London, UK; Fleming Initiative, Institute of Global Health Innovation, Imperial College London, London, UK; Helix Centre, Institute of Global Health Innovation, Imperial College London, London, UK; Fleming Initiative, Institute of Global Health Innovation, Imperial College London, London, UK

## Abstract

Antimicrobial resistance (AMR) is a significant global health threat, yet current efforts to engage young people on this issue remain insufficient despite their central role in shaping future antimicrobial stewardship. This editorial examines the need to rethink AMR education within secondary schooling, arguing for a shift from narrowly science-based instruction towards more holistic, interdisciplinary approaches that address the individual and societal drivers of AMR. A comprehensive review of the national curriculum and examination board specifications in England identified inconsistent and often superficial AMR coverage. Relevant content is largely confined to biology curricula, with minimal integration across subjects such as citizenship and geography, thereby missing clear opportunities to contextualize AMR as a societal, ethical, and behavioural challenge. The Fleming Education Project seeks to address this gap by developing an evidence-based educational intervention, co-designed with young people, to make AMR relevant, actionable, and personally meaningful. By embedding AMR learning and stewardship across diverse subjects and leveraging existing curricular entry points, the project aims to equip young people with the knowledge, attitudes, and behaviours required to prevent the spread of AMR from an early age, empowering them to become future champions of antimicrobial stewardship.

Antimicrobial resistance (AMR) represents one of the most pressing public health challenges of the twenty-first century, driven largely by the misuse and overuse of antibiotics in both healthcare and community settings. Without effective antibiotics, modern medicine stands on precarious ground: routine surgical procedures, cancer therapies, and the treatment of routine infections could once again become life-threatening.^1^ Considerable global attention has been directed towards strengthening AMR surveillance systems, stimulating research and development, and advancing policy frameworks to regulate antibiotic use across human, animal, and environmental health.^2^ Yet, engaging the public through educational initiatives targeted at the root behavioural causes of AMR, arguably the most sustainable and cost-effective route to mitigating AMR, remains underprioritized.^3^

Young people today will inherit the responsibility of managing the global consequences of AMR. The UK’s most recent AMR action plan outlines the importance of empowering and equipping children and adolescents to address AMR.^4^ Education serves as a vital mechanism in this regard, as shaping crucial knowledge, skills, and social responsibility around the appropriate use of antibiotics from an early age can lay the foundation for lasting behavioural patterns that slow the spread of resistance. For example, instructing young people on specific behaviours that can slow the spread of resistance, such as minimizing unnecessary antibiotic use, practising effective hygiene, and promoting vital advocacy to support AMR awareness and sustained behaviour change at the population level, holds significant potential for mitigating AMR. Cultivating a generation actively engaged in reducing AMR is increasingly recognized as a key route to safeguarding public health across communities and generations.^4^ Crucially, as resistance outpaces the development of new antimicrobials—a process dependent on prohibitively costly scientific innovation—education offers a comparatively accessible and scalable solution to mitigating the spread of resistance.^4^

For any AMR educational initiative to achieve sustained impact, it must be meaningfully and strategically integrated into the current educational landscape. Successful integration requires a thorough understanding of what is already being taught about AMR in schools, including the locations of relevant topics across subjects, and an assessment of areas where AMR education could be feasibly and successfully integrated. Such an understanding could help pinpoint entry points for enhanced AMR education across subjects, thereby minimizing curricular burden while ensuring that educational interventions are relevant, accessible, and more likely to be adopted by both students and educators.

To this end, as part of a broader programme of work exploring how best to design AMR educational interventions, the Fleming Education Project at Imperial College London undertook a comprehensive review of the current curricular landscape in England, examining where, how, and to what extent AMR is addressed across the national curriculum and examination board frameworks.^5,6^ Specifically, statutory programmes of study at Key Stages 3 and 4 and GCSE (General Certificate of Secondary Education) specifications across the three major examination boards in England (Pearson Edexcel, AQA, and OCR) were searched for educational content relevant to AMR. This process allowed explicit references to AMR to be catalogued and helped identify areas where other associated themes, such as natural selection, immunology, and public health, surface implicitly, thereby highlighting suitable opportunities for integrating AMR-related learning into the broader curriculum. This baseline mapping also afforded a clearer understanding of the diversity and depth of AMR coverage in schools, revealing both concentrations and gaps in its treatment.

What emerged from the analysis was evidence of a curricular landscape marked by inconsistency and missed opportunities for AMR education. Core topics such as the discovery and mechanisms of antibiotics and the bacterial basis of infection were predominantly confined to biology curricula, with considerably less coverage of relevant themes across other subjects. Notably, the review identified a significant gap in addressing the behavioural drivers of AMR in disciplines beyond the sciences, despite clear intersections with subjects like citizenship, geography, and food preparation and nutrition. These subjects offer valuable opportunities to contextualize the everyday behaviours that influence antibiotic use and infection transmission. For example, geography lessons could explore the agricultural and industrial roots of AMR, where the overuse of antibiotics for growth promotion in livestock and poor hygiene practices may lead to the development of resistant bacteria and its transfer to humans through food chains. Citizenship education could examine individuals’ collective responsibility in preventing the overuse and misuse of antibiotics. Similarly, food preparation and nutrition modules could address hygiene and food safety practices that reduce the occurrence and transmission of bacterial infection. Yet, where these themes do appear outside biology lessons, references tend to be fragmented or superficial, preventing students from gaining a comprehensive or practical understanding of the causes and consequences of AMR.

The review also identified superficial references to AMR-related topics across subjects, such as brief mentions of the medical revolution in history lessons or discussions of universal human rights in citizenship studies. This highlighted significant untapped potential for broader, interdisciplinary engagement on AMR. By positioning AMR not only as a scientific issue but also as a societal challenge with behavioural causes and implications, AMR education could be more powerfully contextualized for young people. This could include, for example, helping students understand how individual behaviours impact public health (e.g. overconsuming antibiotics, failing to adhere to antibiotic prescriptions), exploring ethical dilemmas surrounding antibiotics use (e.g. indiscriminate use of antibiotics in farming), and reflecting on the societal responsibilities for curbing resistance (e.g. to safeguard the health of future generations). Although the widely available e-Bug resources have filled the gap for AMR-related educational material within science lessons in UK schools, evidence indicates that they remain underutilized, with low educator awareness of the programme and usage being largely confined to primary schools.^7^ It has also been suggested that e-Bug could be more effectively positioned as a cross-curricular resource given the duplication of AMR-relevant topics (e.g. hand hygiene) across subjects such as home economics and personal and social health education.^8^ Together, these findings underscore the need for more coherent, cross-curricular approaches to embedding AMR education across secondary education.

Recognizing the fragmented and limited coverage of AMR in current educational offerings is a crucial first step towards the development of more effective AMR education interventions. It is clear that confining AMR to scientific instruction alone overlooks the wider societal, ethical, and behavioural drivers of AMR. Intentional cross-curricular integration is therefore essential to highlight the relevance of AMR to daily life, citizenship, and personal responsibility. Indeed, emerging educational models advocate for integrative approaches to education where teaching and learning occur across two or more subjects with explicit connections emphasized, thereby creating a more cohesive learning experience and better equipping students with the competencies needed to become capable and engaged members of twenty-first century society.^9^ Promisingly, meta-analytic findings indicate that integrated STEM education, where science, technology, engineering, and mathematics are taught through unified lessons, produces moderate gains in student achievement.^10^

Overall, the evidence supports a decisive shift: educational interventions on AMR should be carefully aligned with existing curricular frameworks while fostering a more holistic understanding of the issue. The Fleming Education Project will draw meaningfully on these review findings to inform the design and delivery of new educational initiatives targeted at secondary school students in England aged 14–16. These initiatives are intended to be implemented within school settings and integrated into existing national curricula. The project will leverage existing curricular entry points and strategically address key gaps in AMR education—most notably those related to the behavioural roots of AMR, antimicrobial stewardship, and crucial advocacy efforts aimed at mitigating the spread of AMR. This approach aims not only to enhance young people’s scientific literacy but also to cultivate key attitudes and behaviours to safeguard antimicrobial effectiveness for future generations. Ultimately, positioning the classroom as a catalyst for sustainable behaviour change will be essential to confronting the defining public health challenge of AMR.
